# Time of leaving work pregnancy results during COVID-19 pandemic. The MOACC-19 cohort from Spain

**DOI:** 10.1186/s12889-023-15357-9

**Published:** 2023-03-07

**Authors:** Javier Llorca, Trinidad Dierssen-Sotos, Eugenio Carrasco-Marín, J Lorenzo Guerra-Díez, Carolina Lechosa-Muñiz, María Paz-Zulueta, Inés Gómez-Acebo, María J Cabero-Perez, the MOACC group

**Affiliations:** 1 Universidad de Cantabria- CIBER Epidemiología y Salud Pública (CIBERESP), Santander, Spain; 2 Universidad de Cantabria-IDIVAL-CIBER Epidemiología y Salud Pública (CIBERESP), Santander, Spain; 3 IDIVAL-Instituto de investigación sanitaria Valdecilla, Santander, Spain; 4 Hospital Universitario Marqués de Valdecilla, Santander, Spain; 5 Hospital Universitario Marqués de Valdecilla-Universidad de Cantabria, Santander, Spain; 6 Universidad de Cantabria- GRIDES-IDIVAL, Santander, Spain; 7 Facultad de Medicina, Universidad de Cantabria, Santander, 39792 Spain

**Keywords:** COVID-19, Pregnancy, Leave work, Low weight at birth, Pregnancy control

## Abstract

**Background:**

COVID-19 pandemic has changed the way pregnancies have been controlled as well as working conditions. In countries with paid leave of work, leaving earlier has been a relevant measure for controlling the pandemic. No study has been published on factors associated with earlier leaving work in pregnancy and the consequences it could have on pregnancy outcomes.

**Objective:**

We aimed to identify woman and pregnancy characteristics associated with leaving work earlier and its consequences on pregnancy results. Method: A cohort study was carried out in Cantabria, Northern Spain, including 760 women who were pregnant in 2020 and were working at the beginning of their pregnancy. Data on pregnancy characteristics and results were obtained from medical records and gestational age at leaving work was self-reported. In a logistic regression analysis, leaving work before 26th week of pregnancy was the main effect variable.

**Results:**

Several factors were associated with lower probability of leaving work before 26th week, including university studies (OR = 0.49, 95% CI: 0.36, 0.68), having presential work (OR = 0.57, 95% CI: 0.40, 0.81), women born in non-European countries (OR = 0.55, 95% CI: 0.30, 1.01) and non-smokers (OR for smokers = 1.79, 95% CI: 1.12, 2.87). Neither type of delivery, gestational age at delivery nor other pregnancy results were associated with the gestational age of leaving work.

**Conclusion:**

Several pregnancy and women characteristics were associated with leaving work earlier in the COVID-19 pandemic, although it was not associated with any pregnancy outcome.

## Background

COVID-19 pandemic has changed the way of controlling pregnancy, including teleconsultation [1], more home birth [2, 3], more usage of pain killers and lower rate of episiotomy [2] than in the pre-pandemic period. Pregnant women also reported high rates of unmet need to communicate with a health professional during the lockdown [4] smaller number of antenatal consultations [5, 6] and other disruptions in clinical quality standards [7]. By the other hand, lower rates of caesarean Sect. [8], preterm birth [9]and low weight at birth [8] have been reported during the pandemic in developed countries.

Availability of paid sick leave had been associated with lower probability of attending work when having symptoms compatible with COVID-19 in general (i.e., pregnant or not) workers [10] and has been considered a useful tool for controlling the COVID-19 pandemic [11, 12].

The International Labour Organization (ILO) standards establish a minimum duration of maternity leave of 14 weeks, which they recommend extending to at least 18 weeks to ensure an adequate recovery time for the mother before returning to work [13]. However, there are significant differences in the way maternity leave policy is applied in EU countries [14]. In Spain, the length of paid maternity leave is 16 weeks, but it is only mandatory to take six uninterrupted weeks immediately after delivery. In contrast, more than half of the 28 EU countries have a mandatory maternity leave period prior to birth [15]. In Spain, financial support is also available in the event of occupational risk during pregnancy, which can only be applied for if it has not been possible to change the position to another more appropriate one given their situation.

The Spanish population has universal access to a National Health Service that guarantees the prenatal care for all pregnant women. The effectiveness of preventive care during pregnancy is reflected in its maternal and child indicators. The infant mortality rate in Spain (2.6 deaths per 1,000 live births) is one of the lowest among OECD countries[16]. The perinatal mortality rate, the most useful indicator for evaluating maternal health care in developed countries, is below the EU average (4.5 deaths per 1,000 births vs. 5.2 deaths per 1,000 births) and has experienced a sharp drop of 41% between 1990 and 2017 [17, 18].

The pandemic could have facilitated the obtention of leaving work out of pregnancy or other conditions; to our knowledge, however, no study has been carried out to analyse the effect of time of leaving work on pregnancy results during this period. In addition, the effects of the prenatal maternity leave on the health of mothers and child has been scarcely studied [19, 20].

The main goal of this article is to show the changes in the time of leaving work during pregnancy, the factors associated with leaving work early and its effects on pregnancy outcome, in a cohort of pregnant women assembled in Spain in 2020.

## Methods

### Setting and population

The **MO**ther **A**nd **C**hild **C**OVID-**19** cohort (MOACC-19) was assembled in 2020 to study SARS-CoV-2 infection in pregnant women and their children. Its main characteristics have been described elsewhere [21, 22]. In brief, the cohort began on 26th May 2020 and is formed by three subcohorts, all recruited at University Hospital Marques de Valdecilla (HUMV), Santander, Spain. Subcohort 1 was retrospectively recruited with women delivering from 23rd March to 25th May, 2020. Subcohort 2 was prospectively recruited among women delivering from 26th May, 2020 on. Women in subcohorts 1 and 2 must have been tested for SARS-CoV-2 infection via PCR on the day of admission for delivering. Subcohort 3 was prospectively recruited with women attending their 12th -week of pregnancy control at HUMV obstetrics surgery from 26th May on. They were all tested for SARS-CoV-2 infection via PCR at recruitment. Women in each subcohort were differently exposed to the pandemic and could have taken different protective approaches. Women in subcohort 1 were exposed to the pandemic in the last two months of pregnancy, at the most, so their possibility of taking especial protective measures -as leaving work early- was scarce. Women in subcohort 2 were exposed to the pandemic in their 3rd and, possibly, 2nd trimesters of pregnancy, which coincided with the first pandemic wave. They could have followed stay-at-home orders, move their work to non-presential and advance their leaving work. Finally, women in subcohort 3 were exposed to the pandemic in most of their pregnancy, although their 2nd and 3rd trimester mainly came about through the gap between the first and the second pandemic waves.

### Information and source of data

Information on pregnancy control and delivery was obtained from medical records. It included age, parity, nationality (further classified as European / Non-European), body mass index before pregnancy, gestational weight gain, number and sex of newborns, type of delivery (eutocic, instrumental or Caesarean section), gestational age at delivery, height at birth (later classified as over or under the 10th percentile according to Carrascosa et al. [23], weight at birth (later classified as lower than 2500 g, 2500–3999 and 4000 g or more), Apgar score at 1 and 5 min, pH at birth, neonate feeding at hospital discharge (exclusive breast feeding, artificial formula or mixed breast feeding + formula) and neonatology admission.

Data regarding tobacco or alcohol consumption in pregnancy, educational achievement (classified as primary, secondary, vocational training and university studies), working status (actively working, redundant or student), presential or non-presential work in pregnancy, week of leaving work out of pregnancy were self-reported by the women via personal interview with a midwife. Time of leaving work was initially classified in four categories: 18th week or before, 19th -25th week, 26th -32nd week and 33rd week or later. For analysis requiring dichotomic characterization (e.g., logistic regression), we reclassified time of leaving work as < 26th week / ≥26th week.

### Statistical analysis

This analysis is restricted to women reporting active working in pregnancy and for whom data on delivery are available. The association between woman and pregnancy characteristics associated with time of leaving work was studied with chi-squared test; p value for trend was estimated from Goodman-Kruskal gamma and its asymptotic standard error. A logistic regression analysis was carried out to quantify these associations, using time of leaving work as dependent variable; its results are provided as odds ratio (OR) with 95% confidence interval (CI). A multivariable logistic regression model was built with the variables associated with time of leaving work.

The association between pregnancy outcomes and time of leaving work was studied with chi-squared test; p value for trend was obtained from Goodman-Kruskal gamma test, as explained before. In the logistic regression analysis, time of leaving work was used as regressor and pregnancy outcomes as dependent variable, as leaving work occurred before pregnancy ending. Multivariable logistic regression was carried out adjusting for the woman and pregnancy characteristics found associated with time of leaving work.

No imputation was carried out for missing data. All statistical analyses were performed with the package Stata 16/SE (StataCorp, College Station, Tx, USA).

### Ethics statement

This study was approved by the committee for ethics in research of Cantabria (CEIm Cantabria, record 2020.174). Two different informed consents—one for the mother and one for the child—were signed by the mother before being admitted to the study. The study was conducted according to the Declaration of Helsinki (last update of Fortaleza) and the European Union regulation 2016/679 for the protection of persons regarding the processing of personal data. Before signing the informed consent each potential candidate received information about it from the researchers. The informed consent document included the right to withdraw from the study at any time during the follow-up. To guarantee the privacy and confidentiality of the information obtained, two sets of data were generated:

1) an anonymized main database in which a numerical code was assigned to each participant. This database gathered all the information collected in this study from the participants.

2) a secondary database, only accessible by the study’s data manager, with the identification data of the participants together with their identification code in the main database.

Both bases were stored in encrypted form.

## Results

Out 896 women reporting they were actively working in pregnancy, data on delivery were available for 771. Of them, 760 informed on the week they left work, and so they were included in this analysis. Their description appears in Table 1. Most women were between 35 and 39 years old (n = 312, 40.6%) and 25–34 years old (n = 361, 46.9%), and were born in European countries (706, 93.4%). Out of 50 women born out of Europe, 44 were born in Southern America, 3 in Africa, 2 in Asia and 1 in Centre America. More than 50% women had university studies (408, 53.1%). 5% deliveries were premature (i.e., gestational age at delivery lower than 37 weeks), 5.7% had low weight at birth (i.e., weight lower than 2500 g) and 26.3% required some instrumentation, whether Caesarean Sects. (119, 18.6%) or other instrumentation (49, 7.7%). Seventy-one neonates required admission in the neonatology unit (9.6%).

**Table 1:** Description of the women included in this analysis

Variable	Category	n (%)
**Subcohort**	1st	200 (27.0)
	2nd	253 (34.1)
	3rd	288 (38.9)
**Age at recruitment**	< 25 years	12 (1.6)
	25–34 years	355 (48.4)
	35–39 years	307 (40.5)
	≥ 40 years	84 (11.1)
**Nationality**	European	706 (93.4)
	Non-European	50 (6.6)
**Educational level**	Primary	77 (10.2)
	Secondary	40 (5.3)
	Vocational training	238 (31.4)
	University	403 (53.2)
**Smoking in pregnancy**	No	681 (89.6)
	Yes	79 (10.4)
**Alcohol consumption in pregnancy**	No	740 (97.4)
	Yes	20 (2.6)
**Week of leaving work**	< 19	178 (23.4)
	19–25	166 (21.8)
	26–32	207 (27.2)
	≥ 33	209 (27.5)
**Type of delivery**	Eutocic	467 (73.8)
	Instrumentalized	49 (7.7)
	Caesarean section	117 (18.5)
**Presential work during pregnancy**	No	362 (63.7)
	Yes	206 (36.3)
**Parity**	1	359 (56.1)
	2	239 (37.3)
	≥ 3	42 (6.6)
**Gestational age at delivery**	< 34 weeks	14 (1.8)
	34 < 37 weeks	25 (3.2)
	≥ 37 weeks	721 (94.9)
**Gestational weight gain**	< 9 kg	115 (19.0)
	9–12.9 kg	244 (40.8)
	13–15.9 kg	108 (18.1)
	≥ 16 kg	131 (21.9)
**Pregestational BMI**	< 20 kg/m2	95 (12.6)
	20–24.9 kg/m2	405 (53.6)
	25–29.9 kg/m2	173 (22.9)
	≥ 30 kg/m2	82 (10.9)
**Type of feeding at hospital discharge**	Exclusive maternal breast feeding	432 (58.4)
	Mixed	173 (23.4)
	Formula	135 (18.2)
**Number of newborns**	Single	741 (98.5)
	Twin	11 (1.5)
**Newborn sex**	Male	388 (51.6)
	Female	364 (48.4)
**Height at birth**	≥percentile 10	704 (92.6)
	<percentile 10	56 (7.4)
**Weight at birth**	< 2500 g	43 (5.8)
	2500–3999 g	672 (89.8)
	≥ 4000 g	33 (4.4)
**Apgar 1’**	≥ 8	708 (94.2)
	< 8	44 (5.8)
**Apgar 5’**	≥ 8	746 (99.5)
	< 8	4 (0.5)
**pH at birth**	≥ 7.2	536 (76.5)
	< 7.2	165 (23.5)
**Neonatology admission**	No	655 (90.2)
	Yes	71 (9.8)

### Relationship between women and pregnancy characteristics and time of leaving work

Tables 2 and 3 report the association between pregnancy factors and time of leaving work. More than 50% women in subcohort 2 left work before week 26th, contrasting with 38% and 42% in subcohorts 1 and 3, respectively (p < 0.001), with crude OR = 1.85 (95% CI: 1.27, 2.71) when compared with subcohort 1. Women with university studies scarcely left work before week 26th (only 35% vs. 55% in women with vocational training [crude OR = 0.49, 95% CI: 0.36, 0.68]). Women with presential work in the pandemic were more likely to leave work after week 26th (crude OR = 0.57, 95% CI: 0.40, 0.81, p = 0.002). Other factors associated with leaving work later were non-European nationality, no smoking in pregnancy and consumption of alcohol in pregnancy, although these three factors have small numbers, so their results should be carefully taken. We did not find association with time of leaving work for parity, pregestational BMI, number of newborns and newborn sex.

**Table 2:** Association between women and pregnancy characteristics and time of leaving work

	Time of leaving work (weeks)							
Women characteristics	< 19	19–25	26–32	≥ 33 [of which ≥ 37]		Total	p value*	p for trend**
**Subcohort**							< 0.001	0.39
**1st**	40 (20.1)	36 (18.1)	79 (39.7)	44 (22.1)	[29 (14.6)]	199		
**2nd**	66 (26.3)	68 (27.1)	50 (19.9)	67 (26.7)	[33 (13.2)]	251		
**3rd**	66 (23.6)	51 (18.2)	69 (24.6)	94 (33.6)	[43 (15.4)]	280		
**Age at recruitment**							0.20	0.65
**< 25**	4 (33.3)	2 (16.7)	3 (25.0)	3 (25.0)	[1 (8.3)]	12		
**25–34**	79 (22.3)	90 (25.4)	93 (26.2)	93 (26.2)	[44 (12.4)]	355		
**35–39**	67 (21.8)	56 (18.2)	96 (31.3)	88 (28.7)	[46 (15.0)]	307		
**≥ 40**	26 (31.0)	18 (21.4)	15 (17.9)	25 (29.8)	[17 (20.2)]	84		
**Nationality**							0.27	0.09
**European**	168 (23.8)	158 (22.4)	188 (26.6)	192 (27.2)	[101 (14.3)]	706		
**No European**	9 (18.0)	7 (14.0)	17 (34.0)	17 (34.0)	[7 (14.0)]	50		
**Educational level**							< 0.001	< 0.001
**Primary**	26 (33.8)	18 (23.4)	21 (27.3)	12 (15.6)	[8 (10.4)]	77		
**Secondary**	9 (22.5)	9 (22.5)	10 (25.0)	12 (30.0)	[6 (15.0)]	40		
**Vocational training**	63 (26.5)	67 (28.2)	70 (29.4)	38 (16.0)	[19 (8.0)]	238		
**University**	80 (19.9)	70 (17.4)	106 (26.3)	147 (36.5)	[75 (18.6)]	403		
**Smoking in pregnancy**							0.03	0.003
**No**	150 (22.0)	148 (21.7)	188 (27.6)	195 (28.6)	[102 (15.0)]	681		
**Yes**	28 (35.4)	18 (22.8)	19 (24.1)	14 (17.7)	[6 (7.6)]	79		
**Alcohol in pregnancy**							0.22	0.19
**No**	174 (23.5)	165 (22.3)	199 (16.9)	202 (27.3)	[102 (13.8)]	740		
**Yes**	4 (20.0)	1 (5.0)	8 (40.0)	7 (35.0)	[6 (30.0)]	20		
**Presential work**							< 0.001	< 0.001
**No**	102 (28.2)	76 (21.0)	103 (28.5)	81 (22.4)	[44 (12.2)]	362		
**Yes**	33 (16.0)	40 (19.4)	47 (22.8)	86 (41.8)	[41 (19.9)]	206		
**Parity**							0.85	0.78
**1**	77 (21.5)	88 (24.5)	102 (28.4)	92 (25.6)	[47 (13.1)]	359		
**2**	59 (24.7)	49 (20.5)	67 (28.0)	64 (26.8)	[34 (14.2)]	239		
**3 or more**	12 (28.6)	8 (19.1)	11 (26.2)	11 (26.2)	[9 (21.4)]	42		
**Pregestational BMI**							0.24	0.41
**< 20 kg/m** ^**2**^	23 (24.2)	18 (19.0)	25 (26.3)	29 (30.5)	[19 (20.0)]	95		
**20-24.9 kg/m** ^**2**^	92 (22.7)	97 (24.0)	99 (24.4)	117 (28.9)	[60 (14.8)]	405		
**25-29.9 kg/m** ^**2**^	44 (25.4)	29 (16.8)	52 (30.1)	48 (27.8)	[20 (11.6)]	173		
**≥ 30 kg/m** ^**2**^	18 (22.0)	22 (26.8)	28 (34.2)	14 (17.1)	[8 (9.8)]	82		
**Number of newborns**							0.02	0.33
**Single**	173 (23.4)	165 (22.3)	197 (26.6)	206 (27.8)	[107 (14.4)]	741		
**Twin**	3 (27.3)	1 (9.1)	7 (63.6)	0 (0.0)	[0 (0)]	11		
**Newborn sex**							0.01	0.99
**Male**	79 (20.4)	102 (26.3)	107 (27.6)	100 (25.8)	[51 (13.1)]	388		
**Female**	98 (26.9)	63 (17.3)	97 (26.7)	106 (29.1)	[56 (15.4)]	364		

*p value based on chi-squared test. ** p for trend based on Goodman-Kruskal gamma test

BMI: Body Mass Index

**Table 3:** Women factors associated with leaving work before week 26 of pregnancy (multivariable logistic regression)

Women characteristic	n / N	OR (95% CI)	p	OR (95% CI)*	p*
**Subcohort**					
**1st**	76 / 199	1 (ref.)	-	1 (ref.)	-
**2nd**	134 / 251	1.85 (1.27, 2.71)	0.001	1.49 (0.90, 2.46)	0.12
**3rd**	117 / 280	1.16 (0.80, 1.68)	0.43	1.73 (0.96, 3.10)	0.07
**Age at recruitment**					
**< 25**	6 / 12	1.10 (0.35, 3.48)	0.87	0.86 (0.22, 3.43)	0.83
**25–34**	169 / 355	1 (ref.)	-	1 (ref.)	-
**35–39**	123 / 307	0.74 (0.54, 1.00)	0.05	0.82 (0.56, 1.21)	0.31
**≥ 40**	40 /84	1.21 (0.75, 1.95)	0.43	1.47 (0.80, 2.68)	0.21
**Nationality**					
**European**	326 / 706	1 (ref.)	-	1 (ref.)	-
**Non-European**	16 / 50	0.55 (0.30, 1.01)	0.06	0.49 (0.22, 1.06)	0.07
**Educational level**					
**Primary**	44 / 77	1.11 (0.66, 1.86)	0.70	1.05 (0.54, 2.03)	0.88
**Secondary**	18 / 40	0.68 (0.35, 1.33)	0.26	0.55 (0.22, 1.36)	0.20
**Vocational training**	130 / 238	1 (ref.)	-	1 (ref.)	-
**University**	150 / 403	0.49 (0.36, 0.68)	< 0.001	0.42 (0.28, 0.63)	< 0.001
**Smoking in pregnancy**					
**No**	298 / 681	1 (ref.)	-	1 (ref.)	-
**Yes**	46 / 79	1.79 (1.12, 2.87)	0.02	1.30 (0.69, 2.45)	0.42
**Alcohol in pregnancy**					
**No**	339 / 740	1 (ref.)	-	1 (ref.)	-
**Yes**	5 / 20	0.39 (0.14, 1.10)	0.07	0.23 (0.06, 0.90)	0.04
**Presential work**					
**No**	178 / 362	1 (ref.)	-	1 (ref.)	-
**Yes**	73 / 206	0.57 (0.40, 0.81)	0.002	0.48 (0.28, 0.83)	0.008
**Parity**					
**1**	165 / 359	1 (ref.)	-		
**2**	108 / 239	0.97 (0.70, 1.35)			
**3 or more**	20 / 42	1.07 (0.70, 1.35)	0.85		
**Pregestational BMI**					
**< 20 kg/m** ^**2**^	41 / 95	0.87 (0.55, 1.36)	0.54		
**20-24.9 kg/m** ^**2**^	189 / 405	1 (ref.)	-		
**25-29.9 kg/m** ^**2**^	73 / 173	0.83 (0.58, 1.20)	0.32		
**≥ 30 kg/m** ^**2**^	40 / 82	1.09 (0.68, 1.75)	0.73		
**Number of newborns**					
**Single**	338 / 741	1 (ref.)	-		
**Twin**	4 / 11	0.68 (0.20, 2.35)	0.54		
**Newborn sex**					
**Male**	181 / 388	1 (ref.)	-		
**Female**	161 / 364	0.91 (0.68, 1.21)	0.51		

n: number of women leaving work before week 26. N: total number of women in this group

OR: Odds ratio. CI: confidence interval. *Adjusted for the remaining variables in the column. Parity, pregestational BMI, number of newborns and sex were not included in the multivariable model because of their lack of raw relationship with leaving work

The multivariable logistic regression model supports that leaving work earlier was more frequent in later subcohorts (i.e., those exposed to the pandemic in earlier phases of pregnancy), European women, those without university studies, women without presential work and women reporting having drunk alcohol in pregnancy (Table 3).

### Relationship between time of leaving work and pregnancy outcomes

After analysing type of delivery, gestational age at delivery, gestational weight gained, neonate feeding, height at birth, weight at birth, pH at birth, Apgar score at times 1’ and 5’, and risk of admission at neonatology ICU, we did not find any association with time of leaving work (Table 4). As those pregnancy variables found associated with earlier leaving work in Table 3 could have been confounding factors when studying pregnancy outcomes, we carried out a logistic regression analysis adjusting for them. Its results appear in Table 5, confirming the lack of detectable association between time of leaving and pregnancy outcomes.

**Table 4:** Association between time of leaving work and pregnancy result

	Time of leaving work (weeks)							
Pregnancy result	< 19	19–25	26–32	≥ 33 [of which ≥ 37]		Total	p value*	p for trend**
**Type of delivery**							0.05	0.32
**Eutocic**	102 (69.9)	116 (80.0)	121 (68.4)	128 (77.6)	[66 (74.2)]	467		
**Instrumental**	9 (6.2)	8 (5.5)	16 (9.0)	16 (9.7)	[9 (10.1)]	49		
**Caesarean section**	35 (24.0)	21 (14.5)	40 (22.6)	21 (12.7)	[14 (15.7)]	117		
**Gestational age at delivery**							0.27	0.11
**< 34**	3 (1.7)	5 (3.0)	6 (2.9)	0 (0.0)	[0 (0)]	14		
**34–36 weeks + 6 days**	7 (3.9)	5 (3.0)	8 (3.9)	5 (2.4)	[0 (0)]	25		
**≥ 37**	168 (94.4)	156 (94.0)	193 (93.2)	204 (97.6)	[108 (100)]	721		
**Gestational weight gained**							0.25	0.32
**< 9 kg**	28 (20.1)	29 (21.0)	28 (16.7)	30 (19.6)	[18 (21.7)]	115		
**9-12.9 kg**	49 (35.3)	60 (43.5)	68 (40.5)	67 (43.8)	[34 (41.0)]	244		
**13-15.9 kg**	22 (15.8)	22 (15.9)	31 (18.5)	33 (21.6)	[21 (25,3)]	108		
**≥ 16 kg**	40 (28.8)	27 (19.6)	41 (24.4)	23 (15.0)	[10 (12.1)]	131		
**Neonate feeding**							0.30	0.09
**Breast feeding**	94 (53.7)	97 (60.3)	116 (58.0)	125 (61.3)	[63 (60.0)]	432		
**Mixed**	43 (24.6)	30 (18.6)	49 (24.5)	51 (25.0)	[25 (23.8)]	173		
**Formula**	38 (21.7)	34 (21.1)	35 (17.5)	28 (13.7)	[17 (16.2)]	135		
Height at birth***							0.67	0.29
**≥ percentile 10**	164 (92.1)	151 (91.0)	192 (92.8)	197 (94.3)	[105 (97.2)]	704		
**<percentile 10**	14 (7.9)	15 (9.0)	15 (7.3)	12 (5.7)	[3 (2.8)]	56		
**Weight at birth**							0.38	0.20
**< 2500 g**	9 (5.1)	12 (7.4)	15 (7.4)	7 (3.4)	[4 (3.8)]	43		
**2500–3999 g**	163 (92.1)	144 (88.3)	175 (86.6)	190 (92.2)	[96 (90.6)]	672		
**≥ 4000 g**	5 (2.8)	7 (4.3)	12 (5.9)	9 (4.4)	[6 (5.7)]	33		
**Apgar 1’**							0.09	0.30
**≥ 8**	170 (95.5)	147 (90.7)	192 (93.2)	199 (96.6)	[101 (95.3)]	708		
**< 8**	8 (4.5)	15 (9.3)	14 (6.8)	7 (3.4)	[5 (4.7)]	44		
**Apgar 5’**							1.00	0.88
**≥ 8**	177 (99.4)	161 (99.4)	204 (99.5)	204 (99.5)	[105 (100)]	746		
**< 8**	1 (0.6)	1 (0.6)	1 (0.5)	1 (0.5)	[0 (0)]	4		
**pH at birth**							0.47	0.42
**≥ 7.2**	124 (72.9)	118 (79.7)	143 (75.3)	151 (78.2)	[75 (75.0)]	536		
**< 7.2**	46 (27.1)	30 (20.3)	47 (24.7)	42 (21.8)	[25 (25.0)]	165		
**Neonatology admission**							0.23	0.97
**No**	157 (92.4)	136 (86.1)	180 (91.4)	182 (90.6)	[93 (88.6)]	655		
**Yes**	13 (7.7)	22 (13.9)	17 (8.6)	19 (9.5)	[12 (11.4)]	71		

*p value based on chi-squared test. ** p for trend based on Goodman-Kruskal gamma test

***percentiles based on Carrascosa et al., 2008

**Table 5:** Pregnancy results associated with leaving work before week 26 of pregnancy (multivariate logistic regression)

Women characteristic	n / N	OR (95% CI)	p	OR (95% CI)*	p*
**Type of birth**					
**Eutocic**	218 / 467	1 (ref.)	-	1 (ref.)	-
**No eutocic**	73 / 166	0.90 (0.63, 1.28)	0.55	0.95 (0.59, 1.52)	0.83
**Prematurity**					
**No**	324 / 721	1 (ref.)		1 (ref.)	-
**Yes**	20 / 39	1.29 (0.68, 2.46)	0.44	1.19 (0.51, 2.78)	0.69
**Gestational weight gained**					
**Less than 9 kg**	220 / 483	1.17 (0.78, 1.77)	0.44	1.07 (0.62, 1.85)	0.81
**9 kg or more**	57 / 115	1 (ref.)	-	1 (ref.)	-
**Exclusive natural breast feeding**					
**No**	145 / 308	1.12 (0.84, 1.51)	0.44	1.09 (0.75, 1.59)	0.65
**Yes**	191 / 432	1 (ref.)	-	1 (ref.)	-
**Height at birth**					
**≥Percentile 10**	315 / 704	1 (ref.)	-	1 (ref.)	-
**<Percentile 10**	29 / 56	1.33 (0.77, 2.29)	0.31	1.46 (0.73, 2.93)	0.29
**Weight at birth**					
**2500 g or more**	323 / 717	1 (ref.)	-	1 (ref.)	-
**Less than 2500 g**	21 / 43	1.16 (0.63, 2.16)	0.63	1.17 (0.52, 2.66)	0.70
**Apgar 1’**					
**≥ 8**	317 / 708	1 (ref.)	-	1 (ref.)	-
**< 8**	23 / 44	1.35 (0.73, 2.49)	0.33	0.80 (0.35, 1.81)	0.59
**Apgar 5’**					
**≥ 8**	338 / 746	1 (ref.)	-	1 (ref.)	-
**< 8**	2 / 4	1.21 (0.17, 8.61)	0.85	Model did not converge	-
**pH at birth**					
**≥ 7.2**	242 / 536	1 (ref.)	-	1 (ref.)	-
**< 7.2**	76 / 165	1.04 (0.73, 1.47)	0.84	0.98 (0.63, 1.51)	0.91
**Neonatology admission**					
**No**	293 / 655	1 (ref.)	-	1 (ref.)	-
**Yes**	35 / 71	1.20 (0.74, 1.96)	0.46	1.30 (0.71, 2.39)	0.40

n: number of women leaving work before week 26. N: total number of women in this group

OR: Odds ratio. CI: confidence interval. *Adjusted for subcohort, educational level, nationality, smoking, alcohol consumption, presential work and maternal age at recruitment

**Fig. 1:**
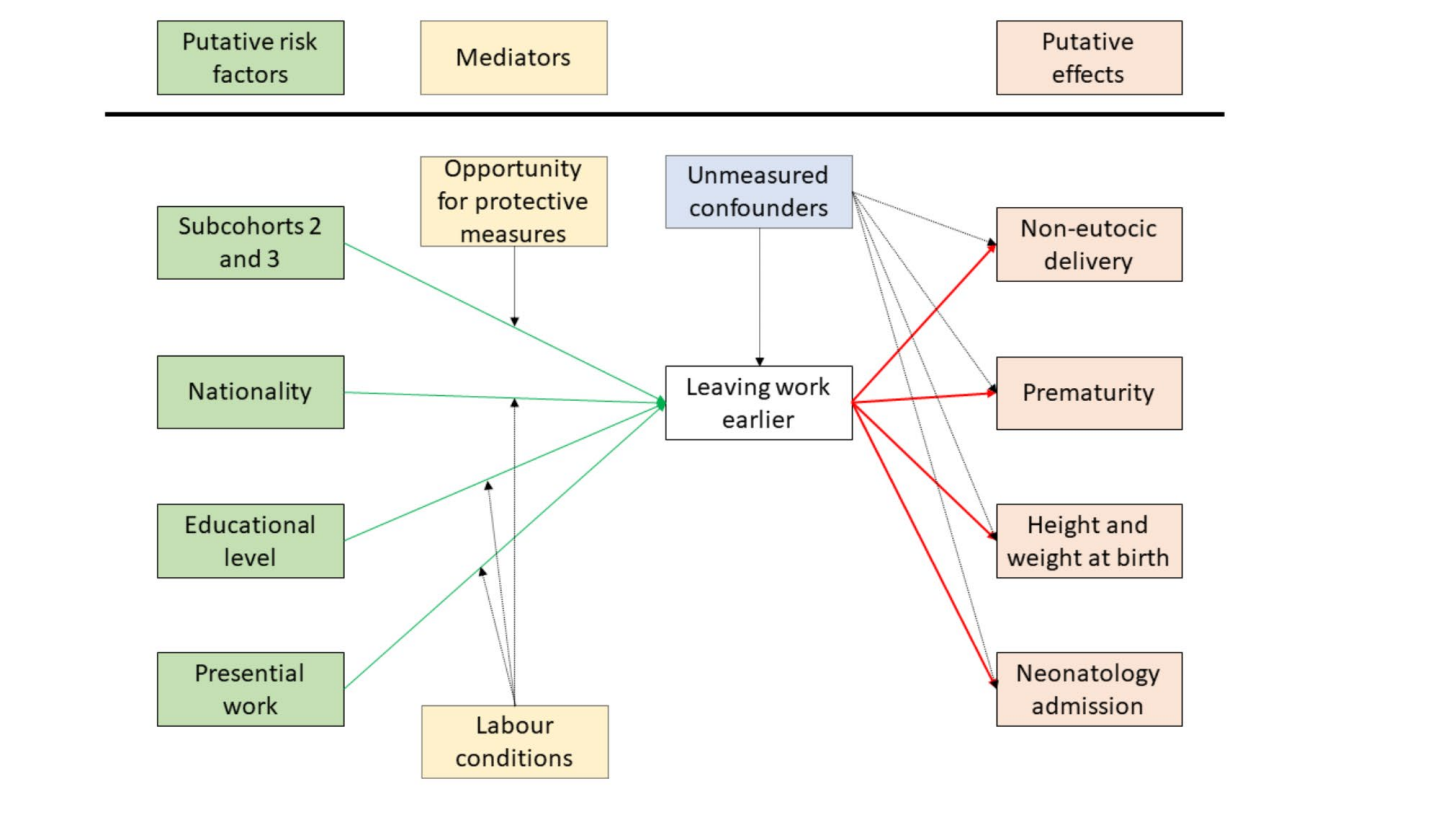
Framework for explanation of factors and outcomes associated to leaving work earlier. Solid rectangles refer to measured variables and dashed rectangles for unmeasured variables. All arrows represent hypothesised associations. Green arrows represent found associations, red arrows stand for unfound associations, dashed arrows symbolize speculated mediation or confounding

Finally, Fig. 1 is centered around the framework of the relationships we could expect to find. We want to remark that the figure does not present what we found but what we could expect to find. Of note, green arrows stand for the relationships we did find (all of them in the “risk factors” side) and red arrows symbolise the expected relationships we did not find (all of them in the “effects” side of the diagram). Dashed rectangles represent unmeasured mediators or confounders.

## DISCUSSION

The main result in this study is that some pregnancy factors, such as subcohort, nationality, educational achievement and presential work, were associated with time of leaving work. Pregnancy outcomes, however, had not such an association.

### Subcohorts and opportunity to take protective measures

Women in subcohorts 2 and 3 left work earlier than women in subcohort 1 did. Of note, subcohort 1 deliveries occurred between 23rd March and 25th May, 2020, very much at the pandemic beginning. Therefore, women in subcohort 1 were aware of the pandemic-associated risks some moment between their 7th and their 9th month of pregnancy. Thus, they had little room to take protective measures in advance. Women in subcohorts 2 and 3, however, were aware of the pandemic early in their pregnancy, so that they had the opportunity to go ahead in protecting them and their children. In addition, the higher incidence of covid-19 in pregnant women observed in the second wave compared to the first one could also explain our findings [24–26] However, one result that bothers us is that women in subcohort 3 did not leave work earlier than women in subcohort 2, as they could have done because they knew about the pandemic in their first trimester of pregnancy. Nonetheless, during their second and third trimesters they had the benefit of the very low COVID-19 incidence between the first and the second waves, which could have prevented them to restrict their outdoors activity, including work.

### Labour conditions in the pandemic and other risk factors for leaving work early

Before the pandemic begun, the Spanish labour market had some especial characteristics: Spain had one of the highest unemployment rates in developed countries (13.8% at the end of 2019), especially affecting younger (27.7% in people aged 20–24) and women (15.6%) [27] and high temporarily rates as well (24.2% in Spain vs. 13.5% in the European Union in 2020) [28]. As the pandemic evolved, the measures enforced by the authorities further limited regular work. On 14th March 2020, a stay-at-home order was approved and a more restrictive confinement was imposed on 29th March 2020; non-presential work was prescribed. When the lockdown was softened, the Spanish Government involved itself in the labour market with the so-called ERTE (abbreviation of the Spanish “employment temporary regulation expedient”), meaning that the employers were allowed to send their employees home for an indeterminate period while being paid by the Administration. About 1.2 million people had been included in ERTE on average between April and December, 2020 [29]; they kept their labour rights and were not considered redundant.

The relationships between time of leaving work and several socio-economic conditions (namely, nationality, educational achievement and presential work) were entangled with this labour context. Women born in non-European countries and living in Spain usually belong to less affluent sectors and have access to more precarious and less qualified works [30], which makes them little empowered to take self-protective decisions, such as leaving work earlier. In our sample, actually, the percentage of non-European pregnant women with only primary or secondary studies more than tripled that of European women (46% in non-European vs. 13% in European women, p < 0.001; results not shown). Our results confirmed that non-European women had about half the probability of leaving work before week 26 of pregnancy when compared with European women. A systematic review identified the education of the mother and belonging to an ethnic minority as determinants of inadequate use of prenatal healthcare in high-income countries[31].

Regarding the influence of educational level on maternity leave, the fact that low-qualified women more often than not, access jobs that entail greater exposure to risk situations (such as shop assistants, domestic cleaners,…)[32] which could contribute to a higher frequency of sick leave. The impact of the level of education on the length of maternity leave shows discrepant results. A study carried out in the USA[33] showed that maternity leave was lower in mothers with fewer years of education, whereas a French study found that highly educated women took maternity leave later[34].We found that pregnant women with university studies left work later than women with lower qualification. A post hoc analysis (results not shown) indicated that women with university studies were more frequently in presential work during the pandemic, but their relative delay in leaving work was about the same in both presential and non-presential work. Working conditions for university-qualified women are usually more stable than for non-university educated women[35] so we would expect they were entitled to take self-protective decisions, such as leaving work early, but our results pointed towards the opposite direction instead. This finding could be related, to the fact that the level of education is inversely associated with the use of antenatal care. Women with higher levels of education tend to have a high level of compliance with the recommended prenatal visits [36], which may be associated with a lower perception of risk during pregnancy and, consequently, take maternity leave later.

On the other hand, we could speculate that university-educated women are more independent and able to take decisions on their will, which could well have led them to a delay in time of leaving work, but we have no data to explore this hypothesis.

### Outcomes related to leaving work early in the pregnancy

Finally, our study fails to find evidence of adverse pregnancy outcomes related to the length of prenatal leave. Likewise, studies carried out in EU countries, where paid maternity leave is a woman´s right, found no evidence of significant effects related to prenatal leave on obstetric complications or children’s health at birth[19, 34, 37]. In contrast, several studies developed in USA found a paradoxical negative effect of antenatal leave that has been explained by the fact that in this country, where paid maternity leave is not widely available, only women with health issues stop working before delivery [20].

Our study has some limitations. Firstly, this study was carried out in a situation far from ideal. Clinical researchers were periodically overwhelmed by clinical work, while non-clinical researchers were compelled to stay at home for some terms. By other hand, in the first wave of the pandemic, hospitals were considered for many patients as especially risky places, which made some women to avoid visit them. All of that resulted in less close researcher – woman relationship and eventually in missing some data. Secondly, our information on labour conditions, stay-at-home orders or temporary regulations of employment is contextual, meaning that we know the rules and the periods, but we do not know how and in what period those rules affected each woman. This fact prevented us to measure the mediators we are speculating with. Thirdly, the above-mentioned peculiarities of the Spanish labour market led to an important shadow economy, which accounted for 20.7% of the Spanish GDP in 2018 [38]. Women in informal economy usually belong to less favoured socio-economic status, have lower educational achievement and have limited labour rights, specifically they do not have the right to be paid after leaving work. Our study only refers to women working in formal economy; thus, it cannot be applied to women in the informal one, which limits the external validity of our results. Finally, to assess the impact of leaving work earlier on the mother and newborn health, we have focused on collecting information related to adverse perinatal outcomes, but we have not included information about mental health. This is a relevant aspect given that the pandemic has had a high psychological impact on the most vulnerable groups such as pregnant women [39] and it is possible that leaving work earlier could reduce it.

Our study has also some strengths. It was carried out in a tertiary hospital of the Spanish public health system, which has universal coverage; its obstetric services are easily accessible without payment, so we do not expect accessibility to be a source of bias. Secondly, most deliveries in Cantabria (the region were the study was carried out) during non-pandemic period occurred in the HUMV and, during the first pandemic wave, all deliveries were concentrated in that hospital.

Summarizing, we found that several socio-economic factors were associated with leaving work earlier in pregnant women during the first year of the COVID-19 pandemic. Differences in the time of leaving work were unrelated with any pregnancy outcome. These findings support the safety of maintaining the current Spanish regulation on maternity leave, which does not impose mandatory antenatal leave in the absence of pathology during pregnancy. Leaving the decision up to the mothers.

Further research is needed to disentangle the complex relations between the associations we found and stay-at-home orders and other public activity restrictions enforced at the pandemic beginning.

## Data Availability

All studies funded by ISCIII in the COVID call for grants should share their data via “Registro ISCIII-COVID19”, which is a repository ruled by the funding institution. This repository is not publicly available yet, although our data were timely sent. Therefore, we have no control on the time it will be publicly available. In the meanwhile we would share our data with other researchers upon reasonable request to the corresponding author.
